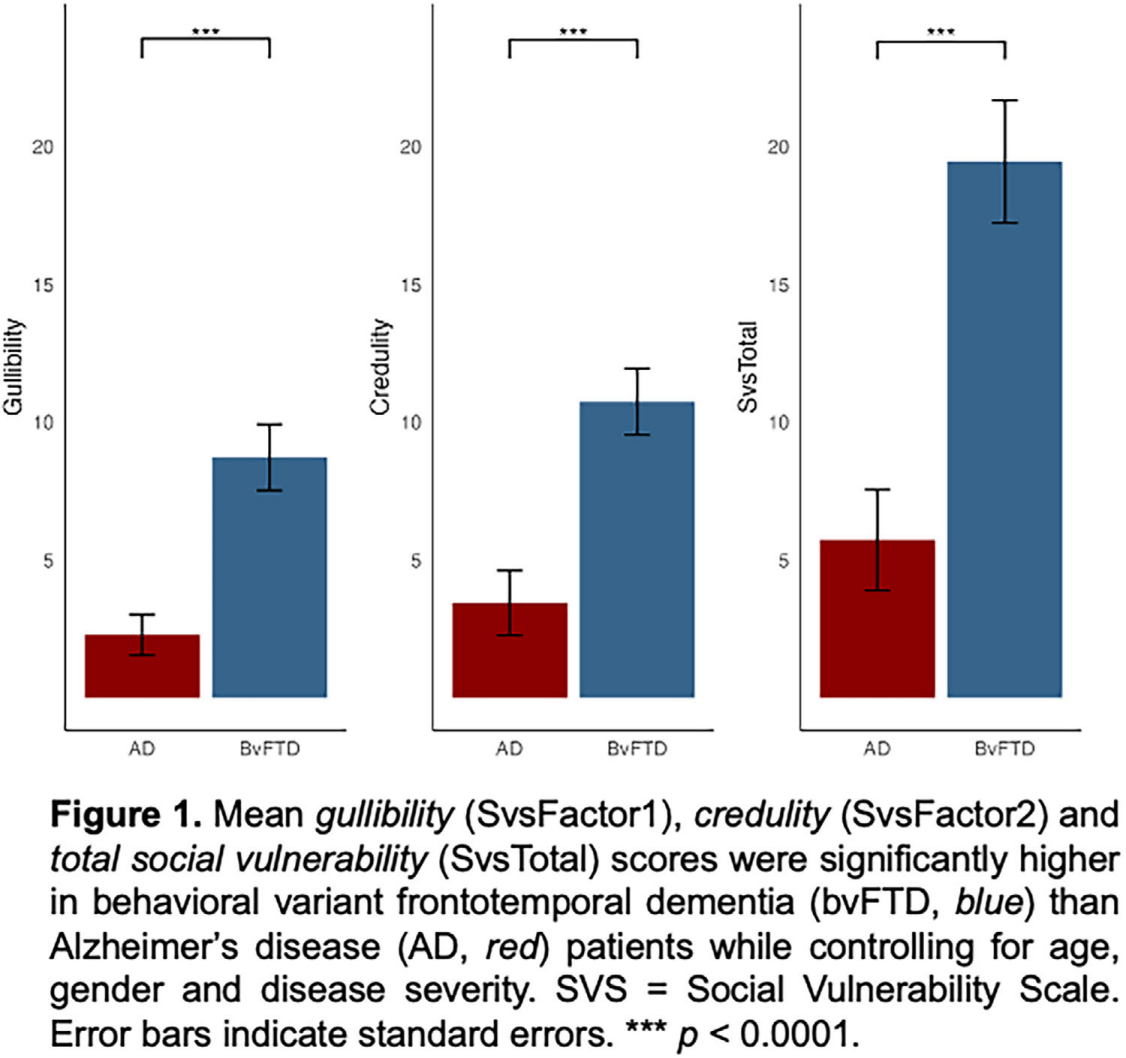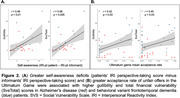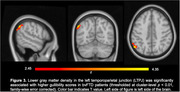# Socioemotional dysfunction relates to financial exploitation vulnerability in Alzheimer’s disease and behavioral variant frontotemporal dementia

**DOI:** 10.1002/alz.089299

**Published:** 2025-01-03

**Authors:** Jayden J Lee, Jerica E Reeder, Tony X Phan, Lindsey C Keener, Siyi J Wang, R. Ryan Darby

**Affiliations:** ^1^ Vanderbilt University Medical Center, Nashville, TN USA

## Abstract

**Background:**

Financial exploitation vulnerability (FEV) denotes the risk for falling victim to financial fraud and older adults reportedly lose an estimated $36 billion annually to scams. Socioemotional and cognitive impairments are potential risk factors for FEV in older adults with dementia. The present study examines whether the socioemotional measures of sensitivity to unfairness and self‐unawareness of socioemotional dysfunction and brain atrophy are associated with increased risk for FEV in Alzheimer’s disease (AD) and behavioral variant frontotemporal dementia (bvFTD).

**Method:**

25 AD and 39 bvFTD patients played the Ultimatum Game (UG), a social decision‐making task that assesses participants’ sensitivity to unfairness. FEV was measured by administering the Social Vulnerability Scale (SVS), an informant‐based questionnaire, comprising two factors for FEV (gullibility and credulity) and total financial vulnerability (SvsTotal). We evaluated patients’ unawareness of their own socioemotional dysfunction by administering the Interpersonal Reactivity Index to both patients and their informants and computed difference scores for perspective‐taking (patients’ score minus the informants’ score). Finally, we used voxel‐based morphometry to determine regional atrophy associated with FEV measures.

**Result:**

Informant ratings for gullibility, credulity, and SvsTotal were significantly higher in bvFTD compared to AD patients (*p*<0.0001) while controlling for age, gender and disease severity. Greater acceptance of unfair offers in the UG was associated with higher gullibility (*r* = 0.42, *p*<0.03) and SvsTotal (*r* = 0.40, *p*<0.03) in both groups. bvFTD showed greater deficits in self‐awareness of perspective‐taking capability than AD patients (*p*<0.02). Unawareness of socioemotional dysfunction was associated with higher gullibility (*r* = 0.46, *p*<0.01) and SvsTotal (*r* = 0.56, *p*<0.005) in both groups. Increased gullibility in bvFTD was associated with gray‐matter volume loss in the left temporoparietal junction (LTPJ), a classic theory of mind region (p<0.01, FWE‐corrected).

**Conclusion:**

Our results suggest that FEV is a more severe problem in bvFTD than AD but distinctly related to socioemotional dysfunction in both groups, including impaired self‐awareness and unwillingness to accept unfair offers in simulated social decision‐making tasks. LTPJ atrophy, critical to perspective‐taking, may also increase FEV susceptibility. Both socioemotional dysfunction itself, and a patient’s lack of awareness for their own deficits (i.e., anosognosia) may potentially be an important early risk factor for FEV.